# Study on the Impact and Water Absorption Performance of *Prosopis juliflora* & Glass Fibre Reinforced Epoxy Composite Laminates

**DOI:** 10.3390/polym14152973

**Published:** 2022-07-22

**Authors:** Manoj Kumar Gurunathan, Navasingh Rajesh Jesudoss Hynes, Omar Ali. Al-Khashman, Michael Brykov, Nagasubramoniam Ganesh, Antoaneta Ene

**Affiliations:** 1 Department of Mechanical Engineering, Mepco Schlenk Engineering College, Sivakasi 626005, Tamil Nadu, India; manojkumargmmech@gmail.com (M.K.G.); ganesh041196@gmail.com (N.G.); 2 Department of Environmental Engineering, Faculty of Engineering, Al-Hussein Bin Talal University, P.O. Box 20, Ma’an 71111, Jordan; omarkhashman@yahoo.com; 3 Department of Equipment and Technologies of Welding Production, Zaporizhzhya Polytechnic National University, 69063 Zaporizhzhya, Zaporiz’ka Oblast’, Ukraine; m@brykov.com; 4 INPOLDE Research Center, Department of Chemistry, Physics and Environment, Dunarea de Jos University of Galati, 47 Domneasca St., 800008 Galati, Romania

**Keywords:** *Prosopis juliflora*, glass fibre, hybrid composite, impact strength, water absorption

## Abstract

Current global trends demand the replacement of synthetic fibres with natural fibres in polymeric composites. The present work makes use of Prosopis juliflora, a plant that is a threat to the environment as a partial replacement in a hybrid composite. Individual Prosopis juliflora fibres are added to matrices at ratios of 12, 6, 9 and 8 wt % and glass fibres are added discretely at ratios of 28, 24, 21 and 32 wt % into matrices as well. The composites are prepared with four different combinations and tested in terms of the mechanical benefits and water absorption performance. This work exploits the mechanical advantage of impact energy in addition to producing Prosopis juliflora particles, fibre glass mats, and resin appropriate for structural uses. Water absorption tests are conducted for four different compositions. Among the four samples, sample 3 (9 wt % Prosopis juliflora fibres and 21 wt % glass fibres) has a higher rate of water absorption than the others, although sample 2 (6 wt % Prosopis juliflora fibres and 24 wt % glass fibres) has a lower rate. The difference in the quantity of water absorption between the hybrid composites can be attributed to the weight percentage of fibres. On the other hand, sample 1 (12 wt % Prosopis juliflora fibres and 28 wt % glass fibres) is reported to have absorbed 2.6 J of energy in the impact strength test. The increase in impact strength is attributed to the increase in the weight percentage of glass fibres. A scanning electron microscope is employed to study the fractured surfaces of the composites. This study shows that the developed hybrid composite could be employed in structural and automotive applications because of its improved impact strength and water resistance.

## 1. Introduction

Over the past couple of decades, resource development has largely been dominated by composites, polymers, and ceramics. The scale and scope of implementations of composite materials have steadily expanded, consistently conquering and capturing market opportunities [1,2]. The present problem is to make affordable composites, despite the fact that they have previously demonstrated their value as weight-saving elements. The composites sector is increasingly using a number of cutting-edge production techniques as a response to efforts to develop economically appealing composite materials. It stands to reason, particularly for composites, that the cost barrier cannot be overcome by advancement in manufacturing techniques alone. For composites to be economical in comparison to metals, there must be an integrated effort encompassing design, materials, techniques, equipment, quality management, manufacturing, and even product management [3,4,5]. A new material may be selected for a variety of reasons, including the fact that it is stronger, lighter, or less expensive than traditional materials. Recently, scientists have begun developing composites termed “robotic materials”, which combine sensing, actuation, computation, and communication capabilities. Some of these composites are used in structures including bridges, boat shells, aquatics panels, rally car bodies, showering stalls, bathtubs, storage vessels, and simulated granite and cultivated marble basins and worktops.

In order to improve composite attributes, hybrid composites are created by employing multiple strengthening elements in much the same way as in polymer matrices. It is feasible to realize whether such addition is of benefit or not by incorporating a range of reinforcing components. Additionally, the qualities can be adjusted by weighing the advantages of one element against its drawbacks. Several reinforcing fibres may be incorporated into a matrix to provide a wider range of qualities than that with individual fibre reinforcement. As a result, researchers have increasingly emphasized the hybridization of reinforcement material with organic and inorganic sources. Since a synthetic material’s hybrid properties are regarded to be better than those of plant fibres, such usage in the machinery and construction sectors is unavoidable.

Numerous efforts have concentrated on hybridization as a means of developing the mechanical characteristics of composite materials that reinforced with natural fibre. When integrating different elements to create a synergistic effect via appropriate material development, lighter hybrid composites can be created. The benefits of one element might supplement what is missing in another, and the effectiveness of the hybrid composite is a balanced total of the separate elements. When using a hybrid design, it is possible to obtain beneficial results, such lower energy usage, advances in strength properties, and economic viability, which may not be possible with the use of the components alone.

Unsaturated polyester resin bonded with glass fibre is known as reinforced plastic. In a few cases, epoxides can be utilized instead of polyester resins. Glass fibres can be woven into fabrics or used as a paper-like material. Due to their high structural performance, excellent thermal and electrical conductivity, max strength percentage, fatigue resistance, rigidity, vibration dampening capacity, servicing nature, greater resistance to chemicals and the atmosphere, and non-toxicity, glass fibre-reinforced plastics have seen an increase in use since the middle of the eighteenth century. Fibre-reinforced composites (FRC) have indeed been employed in a wide range of applications, including sports, medicine, and food packaging, as well as in the realms of aviation, automobiles, and ships [6]. Fibre-reinforced composites are made up of a polymer matrix and reinforcements like particles, fibres, or flakes. When compared to a solitary polymer matrix, the additions are indeed significantly stronger in terms of tensile and flexural strength, which improves the mechanical properties of the composite, such as glass-reinforced fibre [7].

Hybridization is such a technique to improve its mechanical properties in addition to expanding their applicability. As a result, researchers are becoming even more interested in natural fibre-reinforced hybrid composites that incorporate one or maybe more kinds of organic reinforcements. Two or more fibres combined with polymer matrices make up hybrid composites. The volume/weight ratio of the fibres, the order in which the fibre sheets are stacked, how the fibres are treated, and the impact of external elements are the key variables impacting the mechanical characteristics of hybrid composites. Chemical methods can strengthen the bond between both the reinforcement and resin at the junction and reduce moisture absorption.

Natural fibre composites are used for a variety of industrial and domestic reasons. The fact that natural fibres are biodegradable, renewable, reasonably priced, and easily recycled is just some of their benefits [8,9]. Poor bonding hinders mechanical qualities, reducing the use of these composites in industrial settings [10]. Many approaches and procedures have been developed to increase bonding, such as the introduction of coupling agents and the surface modification of fibres through chemical treatments [11,12,13].

Southern India and the desert to the west feature significant amounts of the plant Prosopis juliflora. The plant was developed to meet the demand for fuel since it can endure harsh conditions like high temperatures and tropical climates. However, the Tamil Nadu government in India ordered its uprooting in 2017 because the plant, among the most exotic species, was interfering with the growth of other plants. The bark of the plant has indeed been utilized as an organic fabric since it has the highest tensile strength of any natural fibre while having the lowest density [14,15]. Because particle size is inversely proportional to composite strength, the paper focuses on combining a 2-mm-thick E-glass fibre with fine particles of Prosopis juliflora made into a bidirectional sheet, where epoxy is used as the adhesive (HY951) for setting. The innovative intralaminar hybrid composites are evaluated using empirical impact testing that adheres to the ASTM requirements. Furthermore, the surface morphologies of the investigated samples are analyzed using a scanning electron microscope (SEM).

## 2. Material Selection and Characteristics

### 2.1. Powdered Prosopis juliflora

**Figure 1:**
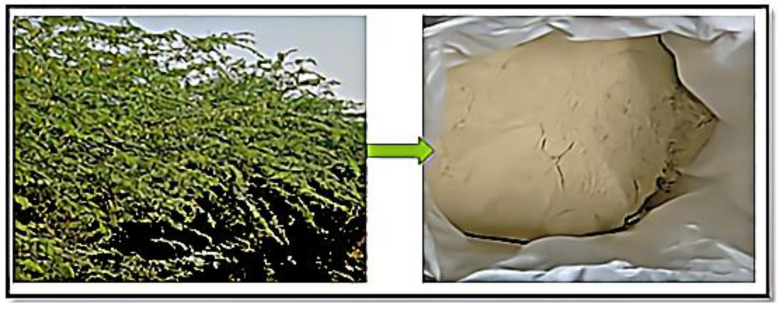
*Prosopis juliflora* powder.

*Prosopis juliflora*, which was previously heralded as a cure-all for the drought-prone regions in Tamil Nadu’s southern districts, is now posing a threat to ecosystems there [16,17]. This perennial plant is a fast-growing tree variant that can withstand arid circumstances and saltwater-dominated soil. It is endemic to Southern and Central America, as well as the Caribbean. Figure 1 displays a Prosopis juliflora powder used in composite preparation. Alkaloids, phenolic compounds, and notably flavonoids are among the phytochemicals abundant in Prosopis plants.

The stem of the *Prosopis juliflora* tree is used to make a powder. To remove the moisture, the stem is cut down and allowed to dry for a few days. The stem is later ground into a powder.

### 2.2. Glass Fibre

**Figure 2:**
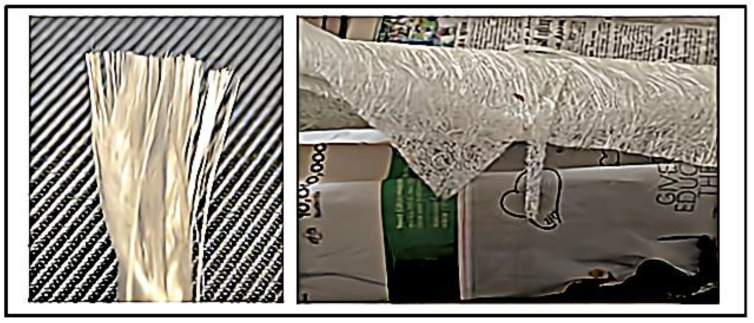
Glass fibre (chopped strand).

Figure 2 depicts the E-glass fibre with the thickness of 2 mm, a substance made up of multiple incredibly tiny glass fibres. Glass fibres are to other fibres like polymers and carbon fibres in terms of the mechanical qualities. Glass fibres are utilized as an enhancer for reinforcement in a variety of polymer-based products in order to create extraordinarily strong and relatively light glass-reinforced plastic, commonly known as “fibre glass”.

### 2.3. Epoxy Resin

**Figure 3:**
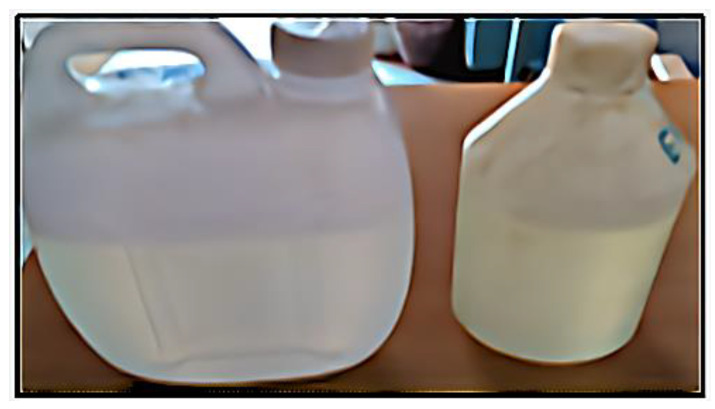
Epoxy resin.

Epoxy resins, as a monomer, can be either high-molecular-weight polymers or reduced pre-polymers. Industrial production processes produce a variety of epoxy resins. The epoxy resin used to construct the composite sample is depicted in Figure 3. HY951 was used as the hardener to make the composite specimen.

Most of the raw materials needed to make epoxy resin now come from petroleum, but some organic sources are slowly becoming commercially available. One example is the organic glycerin used to make chemical reagents [18].

The mechanical characteristics of *Prosopis juliflora*, fibre glass, and epoxy are shown in Table 1. These distinct traits are used in conjunction with the rule of combination to determine the characteristics of the composite specimen.

## 3. Preparation of Composites

The materials which are used in this composite include a chopped glass strand mat, *Prosopis juliflora* powder, and epoxy resin (LY556), along with a hardener (HY917). The sliced glass filament mat was subsequently cut using a die with dimensions of 250 mm × 150 mm × 3 mm. The epoxy and bonding agent were present in matrices here at a 10:1 ratio [19]. The mass of the fibre/resin was determined by the following formula:

Mass of fibre/resin = Volume of die × proportion × density of fibre/resin

**Figure 4:**
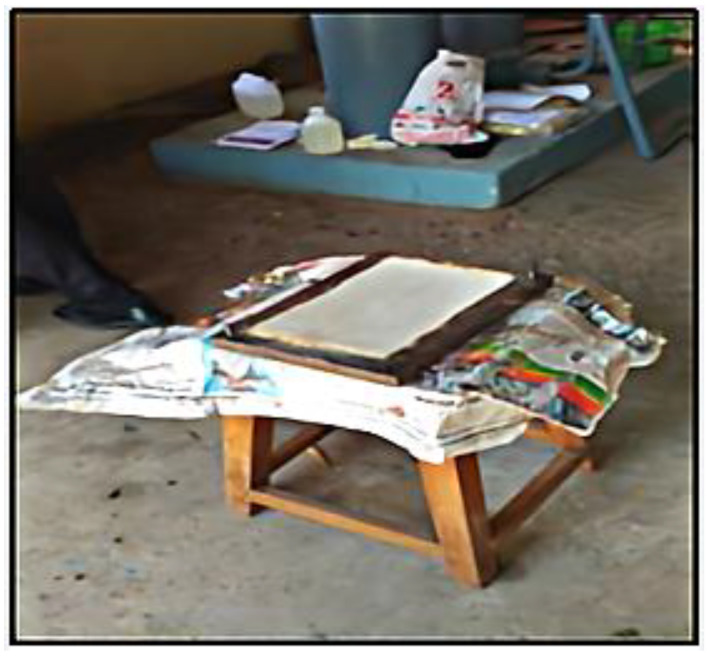
Specimen preparation.

**Figure 5:**
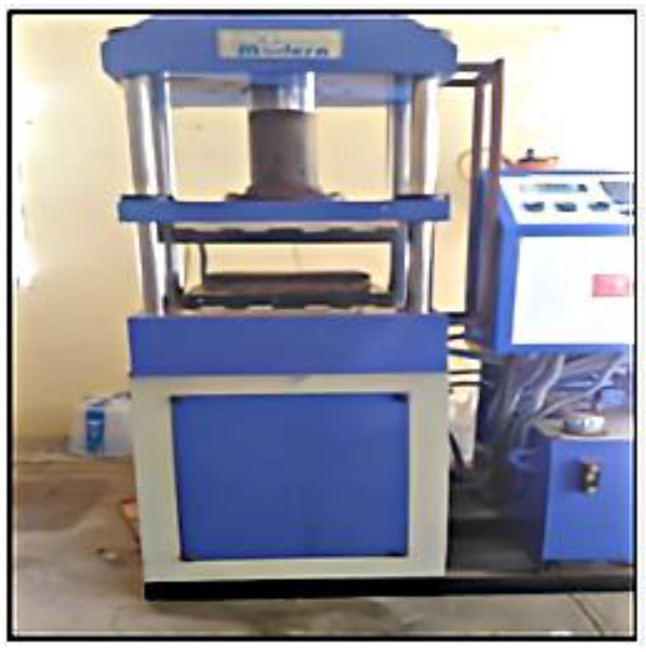
Compression molding machine.

The interlaced glass fibre sheet was cut to the required size. The fibre and matrix would be used in varied weight percentages for the creation of four *Prosopis juliflora* and fibre glass-augmented hybrid composites. The specimen preparation and compression molding machine are shown in Figure 4 and Figure 5.

The epoxy and curing agent were blended in a 10:1 ratio (500 mL epoxy to 50 mL hardener) with a small amount of *Prosopis juliflora*, and the container was constantly swirled to inhibit solidification. The temperature of the epoxy progressively rose during mixing, revealing that bonds started to form. The laminate sheet was waxed on one side before being coated with a glass fibre layer. After the matrix was distributed using a brush and a manual lay-up process, the epoxy was incorporated into fibre glass. To maintain continuity and prevent void development, a roller was applied on top of the resin. After the second layer of the glass fibre was in place, the epoxy resin was poured, and the roller was used to assure perfect placement. On top of that, a laminated sheet with paraffin on the internal surface was added. To prepare the composite, it was retained in the compression molding machine.

The composites are allowed to cure at ambient temperature. The *Prosopis juliflora* glass-reinforced epoxy hybrid composite was cut in accordance with the ASTM guidelines for the experiment. Table 2 displays various fibre and matrix specimen compositions.

**Figure 6:**
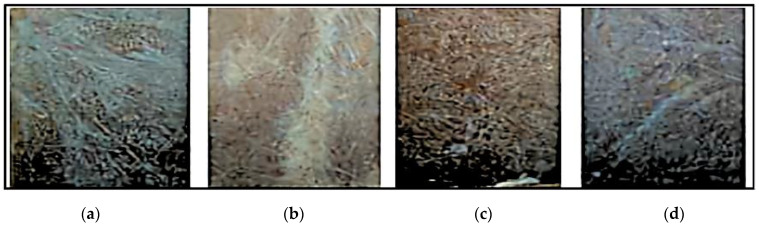
Composite samples. (**a**) Sample 1. (**b**) Sample 2. (**c**) Sample 3. (**d**) Sample 4.

The mass of the fibre/resin for the proportion could be calculated and the materials were taken as per the calculations. The compression molding machine applied a pressure of 50 bar, for which it took 8 h to cure each sample. An extra amount for each matrix was extruded during this pressing operation [20]. Figure 6 depicts the four hybrid composite samples that were developed.

## 4. Experimentation and Results

### 4.1. Impact Test Results

A systematic high strain-rate experiment to measure how much energy is recovered by a material during a fracture is known as a Charpy test, often known as Charpy V-notch assessment. This internal potential was employed to study the air temperature ductile–brittle changeover and assesses the notch toughness. The impact strength and behavior of composite materials were influenced by the kind of matrix, laminate thickness, type of fibre, boundary conditions, lay-up order, and geometric parameters [21,22,23,24,25].

ASTM D256: Length: 65.5 mm and Breadth: 12.9 mm.
Machine: Charpy Impact Testing Machine.

**Figure 7:**
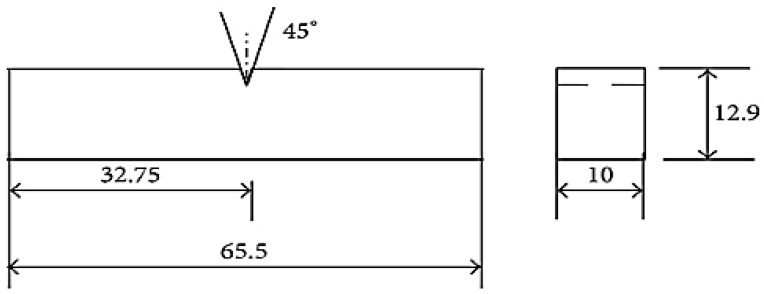
Standard specimen for the impact test.

The standard ASTM size for the impact test is shown in Figure 7. This test is required for the provided application of a car visor. Impact energy absorption is assessed by the use of this test. The energy absorption changes as the proportion of matrix and fibre changes.

**Figure 8:**
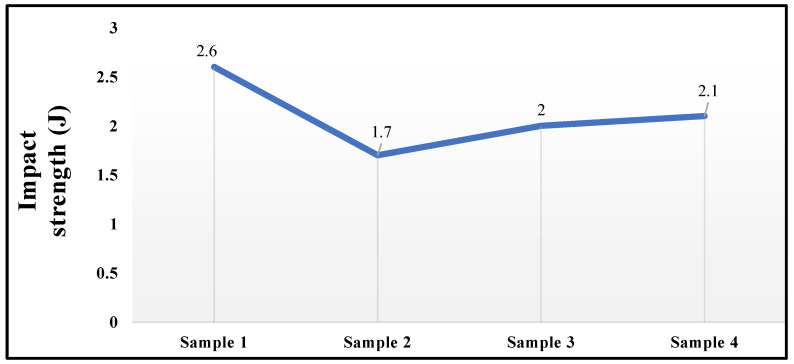
Impact test results.

Figure 8 shows the impact test results. As compared to all the samples, sample 1 gives better impact strength for the applied load. Superior mechanical properties are obtained in the composite sample with more glass fibres. The impact strength of the composites improved as the fibre glass content increased.

### 4.2. Scanning Electron Microscope Results

A scanning electron microscope (SEM) scans a material’s surface with a focused stream of electrons to produce an image of the material. The interaction of the electrons with the sample’s molecules results in a variety of signals that provide details on the sample’s surface topography and chemical makeup. An image is created by combining the received signal with the direction of the electron beam as it is scanned in a raster scan pattern. SEM offers a resolution greater than 1 nm. Samples can be tested in a variety of cryogenic or high temperatures using specialist equipment, as well as in a vacuum condition in a normal SEM or a lower vacuum or wet environment in a flexible pressure or environmental SEM.

**Figure 9:**
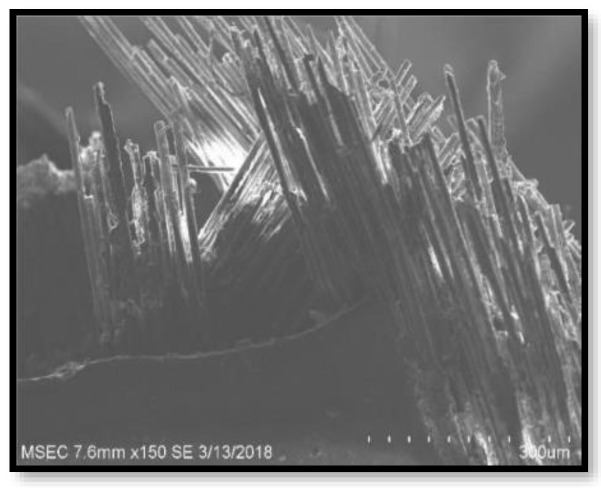
SEM image of Sample 1.

**Figure 10:**
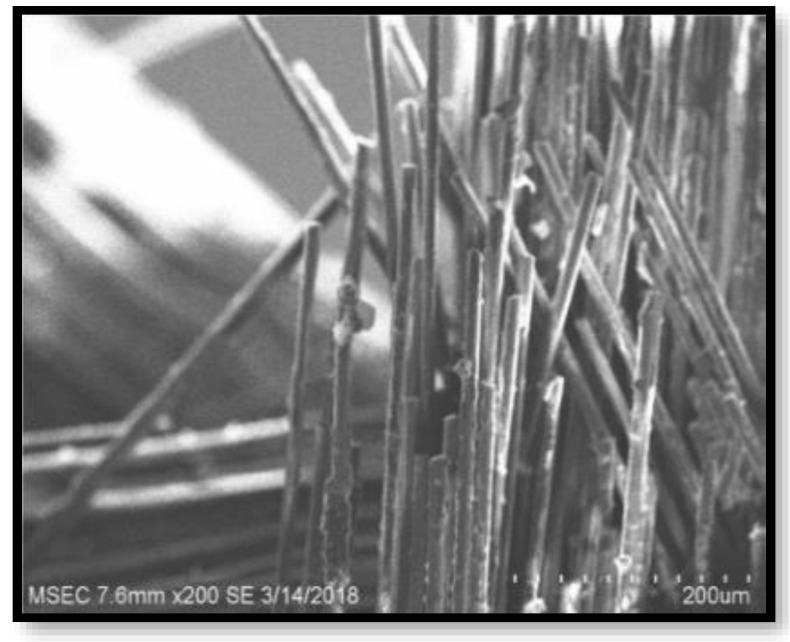
SEM image of Sample 2.

**Figure 11:**
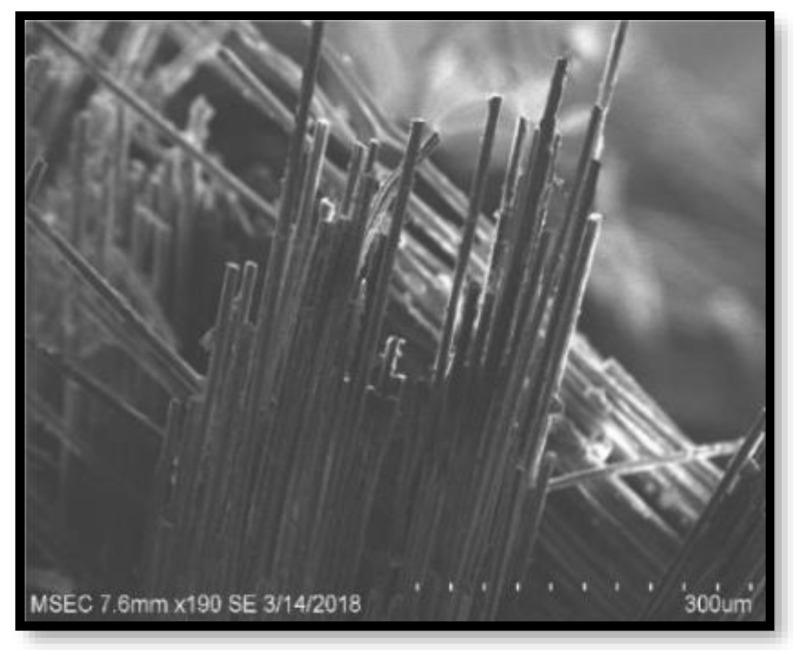
SEM image of Sample 3.

**Figure 12:**
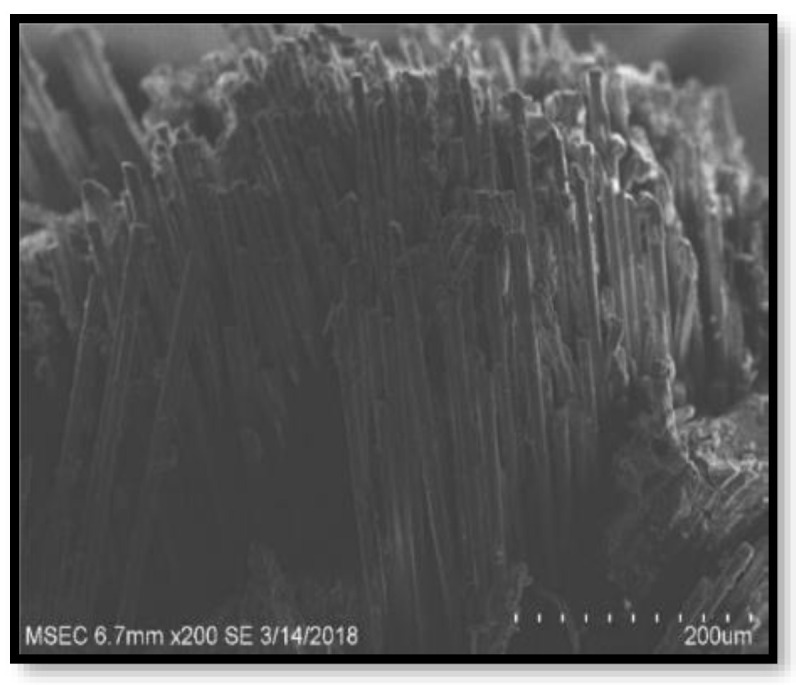
SEM image of Sample 4.

Figure 9, Figure 10, Figure 11 and Figure 12 present the results of SEM examination of the samples to assure an appropriate spectrum of dimensions of the fibres. It is generally presumed that the smaller the fibre size in a hybrid composite, the more it will aid in enhancing the composite’s strength. The size, direction of fibre alignment, voids, interfacial binding, particle scattering, and other elements all influence the composite’s characteristics. In the untested and tested fibre-reinforced specimens, the effect of testing on the internal structure of the composite can be seen. Figure 12 shows a continuous band of fibres oriented in a specified orientation, which will be used for the experiment. The investigated GFRP sample’s SEM micrograph reveals many fibre pull-outs and cracks, as well as the development of voids in the structure due to the loading.

**Figure 13:**
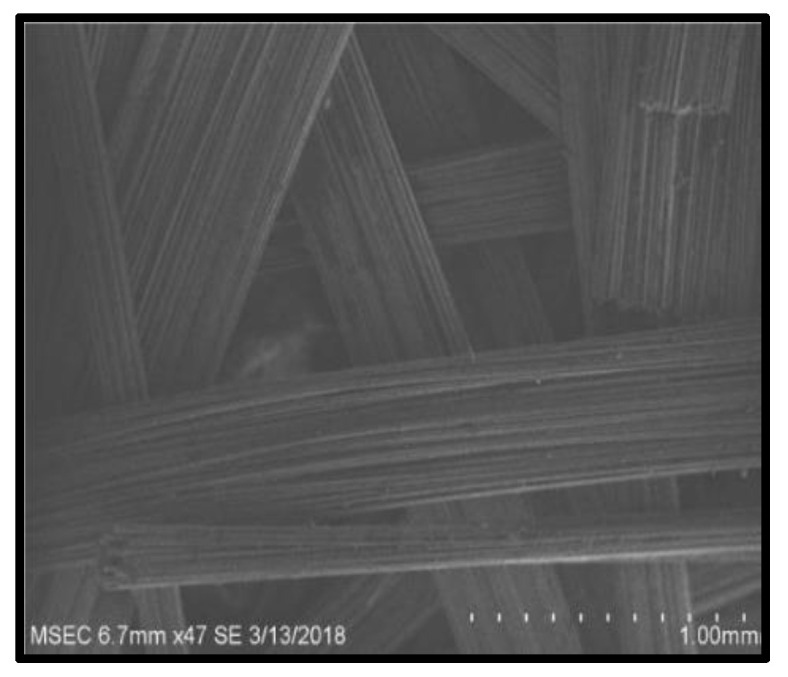
SEM image of glass fibres.

**Figure 14:**
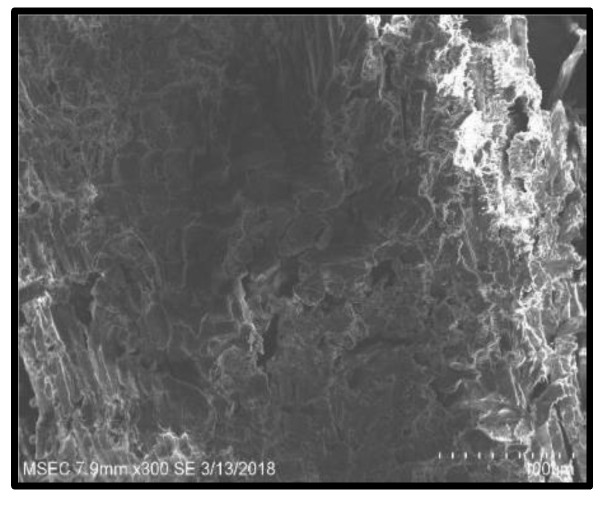
SEM image of *Prosopis juliflora* powder.

The interaction between the matrix and the reinforcement was examined using a scanning electron microscope on the perforated faces of *Prosopis juliflora* fibre epoxy composites. Figure 9, Figure 10, Figure 11 and Figure 12 show samples at 100× magnification, displaying the obtained results. Figure 9 shows that there were fewer fibre pullouts, which increased the mechanical characteristics. SEM images of Glass fibres and *Prosopis juliflora* powder are depicted in Figure 13 and Figure 14. Large portions of the fine fibre glass-reinforced composites showed obvious resistance to the impact force during testing. Sample 1 plainly shows the largest area of resistance to the impact force, since it has the greatest mechanical qualities as compared to samples 2, 3, and 4.

### 4.3. Testing for Absorption of Water

The amount of water absorbed under particular conditions can be compared by the absorption of water. The samples were left to dry out in a furnace at a specific temperature and for a specific amount of time before being chilled in a centrifuge tube for experimental investigation. As soon as the exhibits had cooled, they were examined. The material was then dissolved in water for 24 h or until equilibration at the appropriate temperature, usually 23 °C. Before being weighed, the samples were dried with a lint free cloth.

The rise in the weight percentage in terms of water absorption is expressed as follows:

(1) $$\mathrm{Percent}\;\mathrm{Water}\;\mathrm{Absorption}=\frac{W_{1}-W_{0}}{W_{0}}\times 100$$

where *W*_0_ is the weight of the composite before being submerged in water, and *W*_1_ is the weight of the composite afterwards. For testing, the length was 20 mm and the breadth was 20 mm.

**Figure 15:**
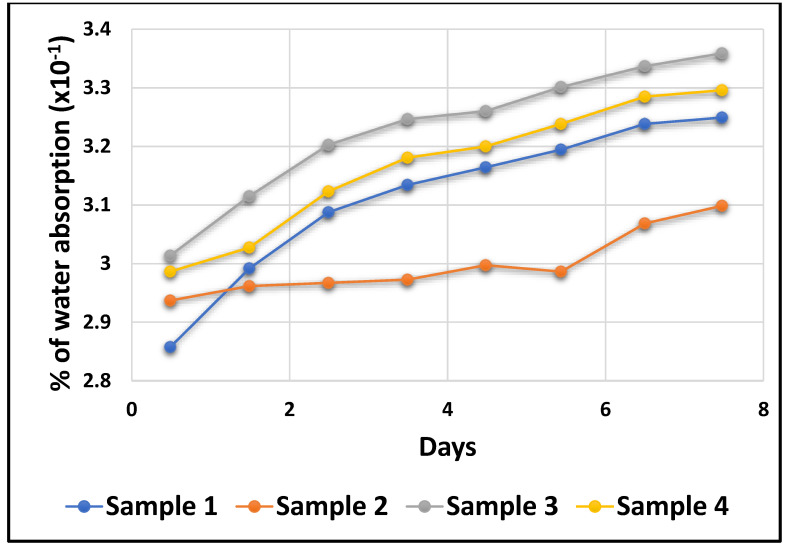
Water absorption test plot.

To comprehend the durability of composites depending on specific field of application, it is essential to investigate the moisture absorption behavior of hybrid composites. Moisture is absorbed primarily by hydrogen bonding at the interface between the fibre and the matrix, as well as by the fibre itself. Figure 15 shows the proportion of water absorption for four distinct proposed samples. Equation (1) was used to calculate the amount of water molecules that penetrated the composite specimens. After a week, the proportion of water absorption increased with immersion time and remained consistent. Figure 15 demonstrates that sample 3’s hybrid composite had the largest moisture content, followed by sample 4’s hybrid composite. The difference between the hybrid composites in samples 3 and 4 can be attributed to the weight percentage of fibres. The sample 1 hybrid composite consumed less water than samples 3 and 4, on average. Higher reinforcing improved water absorption because it enhanced the contact between hydrogen atoms and fibres. Additionally, the proportion of water absorption gradually increased as the immersion period progressed, proving the continuous water absorbing of hybrid composites.

High reinforcement improved water absorption because it enhanced the contact between water molecules and fibres. Additionally, the amount of water absorption increased proportionally as the immersion period progressed, indicating that natural fibre composites continually absorb water. Sample 3 had a high water absorption rate compared to the other samples, while sample 2 had a low absorption rate (Figure 15). *Prosopis juliflora*’s ability to absorb water decreases as its content increases in a matrix. For the fluctuations in the values of the natural fibre content within a matrix, the values are varied in the other samples.

### 4.4. Fabrication of Side Visor

Due to its low cost, ease of manufacturing, reduced density and weight, and growing focus in reprocessing and the impact of materials on the environment, hybrid composites derived from Prosophis julifora have rekindled interest in the automobile sector, such as for the transmission of the stress load between surfaces, where the proposed composite demonstrates acceptable fibre-to-matrix interaction qualities.

**Figure 16:**
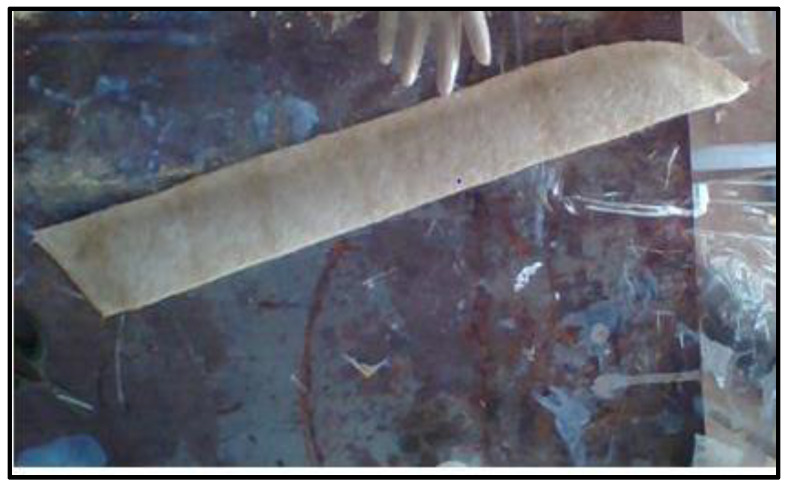
Fabrication of a side visor using composites.

A car side visor made of a composite material is shown in Figure 16. A car side visor is located above the doors of some automobiles, to guard the car from rain or other precipitation in the case of slightly opened windows.

It may also aid in preventing precipitation from entering the interior in the event of an open door, such as rain falling from the roof or reliably from the air. Deflectors are also tailored to sunroofs to deflect wind. As such, this composite can be effectively recommended for use here, particularly in automotive and traditional structural fields.

## 5. Conclusions

In the study, four *Prosopis juliflora*/glass/epoxy resin hybrid composite laminate samples were developed by compression molding, and different experiments on the impact strength and water absorbance were carried [26]. The following conclusions can be made after observation of the tests.

⮚ The impact resistance of sample 1 (12 wt % *Prosopis juliflora* fibres and 28 wt % glass fibres) was approximately 2.6 J. Since this value is larger than that of the other samples, sample 1 was chosen as the material with the highest resistance to impact strength.
⮚ Sample 2 (6 wt % *Prosopis juliflora* fibres and 24 wt % glass fibres) is much more suitable for use in the external parts of automobiles since it has a reduced water absorption capacity.
⮚ When compared to the other samples, sample 1 has a good bonding nature as revealed in the SEM micrograph.

Prosophis julifora glass fibre epoxy composite specimen 1 outperformed traditional GFRP in the comparison tests. This is a result of the improved load distribution and interfacial interaction between the fibre and matrix. The composite also has excellent mechanical characteristics and water absorption due to the absence of pores. Due to the abundance of *Prosopis juliflora* trees, the composite can be used as an outlay option for automobile applications, such as car visors.

## Figures and Tables

**Table 1:** Properties of Materials.

	*Prosopis juliflora*	Glass Fibre	Epoxy Resin
Young’s modulus (E_f_) N/m^2^	30.00 × 10^9^	72.00 × 10^9^	4.00 × 10^9^
Density (ρ) Kg/m^3^	0.580 × 10^3^	2.56 × 10^3^	1.20 × 10^3^
Poisson’s ratio	0.21	0.21	0.40

**Table 2:** Compositions of samples.

	Sample 1	Sample 2	Sample 3	Sample 4
Matrix	60%	70%	70%	60%
Glass	28%	24%	21%	32%
*Prosopis juliflora*	12%	6%	9%	8%

## Data Availability

The data presented in this study are available on request from the corresponding author.
